# Furthering nutrition equity through innovative and empathetic collaborations with the restaurant sector: Examples from Latin American restaurants

**DOI:** 10.3389/fpubh.2023.1058859

**Published:** 2023-02-02

**Authors:** Melissa Fuster

**Affiliations:** School of Public Health and Tropical Medicine, Tulane University, New Orleans, LA, United States

**Keywords:** diet, food environment, Hispanic (demographic), restaurant, health equity, human centered design, community nutrition

## Introduction

Dietary factors are one of the leading causes of preventable death and disability (1). Successful dietary interventions to address this problem can only be successful when food environments support healthy food choices (2, 3). Current efforts to improve local food environments have focused on markets to positively influence at-home food consumption, but there is an increased interest in restaurants as sites for intervention and regulation (4, 5). The focus on restaurants responds to shifts in consumption patterns, where an increased proportion of our food spending has been shifting to foods away from home. In the United States, data from 2021 show that the consumption of foods prepared away from home accounts for 55% of food spending among American households, with restaurant meals occurring at least weekly for two-thirds of adults in the United States (6, 7).

Research addressing foods away from home has highlighted the unhealthy aspects of restaurant offerings (8, 9). In particular, increased consumption of meals from fast-food restaurants has resulted in greater intakes of saturated fat and sodium (10). Public health initiatives and policies to improve food choices at restaurants have included efforts to restrict choice (ex. trans-fat ban law) or guide choice through pricing schemes, point of sale promotion of healthy options, and providing nutrition information, among others (11). Most of these efforts have targeted chain-based, fast-food restaurants. Emerging research in small, non-chain restaurants demonstrates interventions can be successful at increasing the consumption of healthier options, through point-of-purchase promotion of healthy dishes and increasing the availability of healthier options (12). While these efforts are important, there has to be a shift in how food businesses are approached from places of perceived primarily as sites for unhealthy eating to places with the potential for motivating healthful dietary changes. This shift may result in a true partnership with restaurants, with the potential to enhance social opportunities for healthier eating, contending against social norms and perceptions where healthier choices are viewed as restrictive, bland, or plainly not enjoyable (13). These perceptions are an important, yet overlooked barrier, to healthy eating behaviors. Diners prioritize taste and indulgence when dining out and the use of healthy designations actually dissuades customers from ordering these items (13). Labeling food as healthy decreases perceived satiety and influence the perception that healthy items are less filling (14, 15).

## Restaurants as agents for dietary change

Restaurants are an important part of community food environments, affecting local food availability and access (3, 16). Restaurants can serve as vehicles to spread culinary innovations by exposing clients to new ingredients and modes of preparations, with the potential to change perceptions and social norms around eating and cooking—changes that can have a ripple effect on foods eaten at home (17, 18). The culinary sector is increasingly involved in initiatives to motivate healthful eating practices as demonstrated in the “Menus for Change” report produced by the Culinary Institutes of America, which guides how to provide healthier and more sustainable meals (19). Other examples are found among an increasing number of restaurants, such as Alice Waters' Chez Panisse restaurant, Chef Dan Barber's sustainable approach to cuisine, and Chef Jamie Oliver's attention to healthy meals for school children (20, 21). These chefs are promoting menu innovations to bring environmental consciousness to their customers, addressing fashion, health, environmental sustainability, and deliciousness as convergent rather than contradictory values. While these new trends bend toward more healthy and environmentally sustainable offerings, these innovations have largely failed to trickle down to restaurants serving communities experiencing the largest burden of disease from diet-related conditions, as in the case of Hispanic/Latin communities. Latin American restaurants (LARs) are an increasingly important sector. According to the National Restaurant Association, 80% of consumers eat at a restaurant serving ethnic cuisine at least once a month (22). Within these, there are over 120,000 LARs in the United States, most of which are independently owned. Mexican restaurants alone make up 8% of all US restaurants (23, 24).

Cuisines in Latin America and the Caribbean are undergoing a transformation where local restaurants are playing a role in creating new, desirable perceptions of their localities and cuisines, bringing locally grown foods to consumers. Research has mostly documented these transitions in México and Perú (25, 26), but similar trends are also found across the region. Innovations include showcasing fruits and vegetables in a new light, appealing to the origin of the product and sensorial qualities, and highlighting healthy dishes without focusing on health. Restaurant menus are elevating fruits and vegetables to a new light, communicating the importance of fresh, local foods. We can also see the emergence of vegetable-forward dishes, where plant-based components take precedence over animal-sourced products. This is important in Latin/Hispanic diets, as the consumption of meat has been tied to socioeconomic status, given the higher quality of animal protein. In many global contexts, meat is eaten only on a special occasion, with cheaper meats, such as chicken, eaten more frequently in lower socioeconomic strata (27). Trends are changing, where menus were more varied in offerings, not focusing on beef and pork and presenting more plant-forward menus, catering to an increased interest in this type of offering among higher socioeconomic strata (28). We can also find innovative ways to promote potentially healthful dishes, such as framing these within the historic context, and seeking to re-center them as part of the national culinary imaginary. This shows that chefs in Latin America are innovating local and native ingredients to produce more visually pleasing and tasteful dishes. This approach can facilitate healthier eating and better health outcomes if disseminated outside the elite markets that most of these restaurants attract. The dissemination of these practices requires a shift in thinking among public health researchers and practitioners, to view restaurants in a new light, innovating how we approach and view the sector.

The Latin American Restaurants in Action (LARiA) project is an example of the shift. Through this project, I have sought to disseminate emerging healthy eating promoting innovations to restaurants serving Latin/Hispanic communities in the United States. The LARiA Project is a pilot initiative funded by the National Institutes of Health, motivated by a recognition that community ethnic restaurants, as in the case of those serving Latin American cuisines, are crucial, yet untapped sites for healthy eating promotion interventions (11, 12). These restaurants are important institutions in immigrant communities. Aside from serving as a site to reconnect with heritage cultures through cuisines, these restaurants also serve as sites for social interactions and venues for economic opportunity, while also promoting cross-cultural exchanges (18, 29–31).

The project was launched in the summer of 2020, amid the COVID-19 pandemic (32). Working with a multidisciplinary team, we conducted listening sessions with Latin restaurant owners and staff to learn about their ongoing experiences and needs, as well as their opinions and previous engagement to promote healthier choices (33). The participants in these sessions shared a variety of experiences, from restaurants that sought to actively create healthier dishes to those resistant to change, amid perceptions of client demand and notions of cuisine authenticity. From these sessions, we engaged two restaurants in the intensive process of co-designing tailored innovations to promote healthier choices, *via* the application of human-centered design (HCD). HCD required our approach to be taken constructively and experimentally, rooted in the needs and context of the end user, in this case, the restaurants and consumers, to develop bottom-up solutions with built-in buy-in (34). We used traditional public health research methods, such as interviews and environmental assessment, but also engaged in activities to increase our understanding of the contexts we aimed to change *via* immersion activities, placing ourselves in the shoes of key stakeholders (e.g., customers and owners) to gain a deeper understanding of the constraints behind the consumption and provision of healthier choices in these establishments. We collaborated with a designer to facilitate workshops with partner restaurants that resulted in finding a common ground concerning problem definition and potential solutions to test in the field before full implementation. Following this methodology, we successfully worked with two restaurants, resulting in tailored innovations that addressed restaurant-defined problems that increased healthy offerings and promoted existing offerings using messaging that appealed to taste and the historical roots of these offerings, respectively (35, 36). Efforts like these have the potential to make healthier choices not only available and accessible but also desirable.

The LARiA Project is just an example within growing efforts that seek to engage this sector (37–39). This previous work showcases best practices for engagement with the sector, including the importance of active engagement with restaurant owners, through personal, tailored approaches that incorporate members of the sector and community in the research team (39). Yet, this is contrary to the prevalent top-down regulatory approaches that impose stringent standards continue to be at odds with the economic goal of restaurants, especially small, independently owned restaurants, where profit margins are slim and operational burdens are high (33). Moreover, these establishments are often embedded in communities where diet-related health inequities are prevalent, where owners and staff are also part of these communities, seeing and being affected by the same issues we seek to address (30). We need to engage the sector through transdisciplinary approaches that allow for innovations at different levels, seeking a deeper understanding of the restaurant context and the individuals embedded in these contexts.

## Author contributions

MF conceptualized the article structure, wrote, and edited all aspects of the manuscript.
